# Advanced Glycation End Products and Bone Metabolism in Patients with Chronic Kidney Disease

**DOI:** 10.1002/jbm4.10727

**Published:** 2023-02-16

**Authors:** Kélcia R. S. Quadros, Noemi A. V. Roza, Renata A. França, André B. A. Esteves, Joaquim Barreto, Wagner V. Dominguez, Luzia N. S. Furukawa, Jacqueline Teixeira Caramori, Andrei C. Sposito, Rodrigo Bueno de Oliveira

**Affiliations:** ^1^ Nephrology Division, School of Medical Sciences University of Campinas (Unicamp) Campinas Brazil; ^2^ Laboratory for Evaluation of Mineral and Bone Disorders in Nephrology (LEMON), School of Medical Sciences University of Campinas (Unicamp) Campinas Brazil; ^3^ Laboratory of Renal Pathophysiology, LIM‐16, Department of Internal Medicine, School of Medicine University of São Paulo São Paulo Brazil; ^4^ Department of Internal Medicine, Botucatu School of Medicine UNESP São Paulo Brazil; ^5^ Laboratory of Atherosclerosis and Vascular Biology, Cardiology Division School of Medical Sciences, University of Campinas (Unicamp) Campinas Brazil

**Keywords:** ADVANCED GLYCATION END PRODUCTS, BONE METABOLISM, CHRONIC KIDNEY DISEASE

## Abstract

Advanced glycation end products (AGEs) accumulation may be involved in the progression of CKD‐bone disorders. We sought to determine the relationship between AGEs measured in the blood, skin, and bone with histomorphometry parameters, bone protein, gene expression, and serum biomarkers of bone metabolism in patients with CKD stages 3 to 5D patients. Serum levels of AGEs were estimated by pentosidine, glycated hemoglobin (A1c), and N‐carboxymethyl lysine (CML). The accumulation of AGEs in the skin was estimated from skin autofluorescence (SAF). Bone AGEs accumulation and multiligand receptor for AGEs (RAGEs) expression were evaluated by immunohistochemistry; bone samples were used to evaluate protein and gene expression and histomorphometric analysis. Data are from 86 patients (age: 51 ± 13 years; 60 [70%] on dialysis). Median serum levels of pentosidine, CML, A1c, and SAF were 71.6 pmol/mL, 15.2 ng/mL, 5.4%, and 3.05 arbitrary units, respectively. AGEs covered 3.92% of trabecular bone and 5.42% of the cortical bone surface, whereas RAGEs were expressed in 0.7% and 0.83% of trabecular and cortical bone surfaces, respectively. AGEs accumulation in bone was inversely related to serum receptor activator of NF‐κB ligand/parathyroid hormone (PTH) ratio (R = −0.25; *p* = 0.03), and RAGE expression was negatively related to serum tartrate‐resistant acid phosphatase‐5b/PTH (R = −0.31; *p* = 0.01). Patients with higher AGEs accumulation presented decreased bone protein expression (sclerostin [1.96 (0.11–40.3) vs. 89.3 (2.88–401) ng/mg; *p* = 0.004]; Dickkopf‐related protein 1 [0.064 (0.03–0.46) vs. 1.36 (0.39–5.87) ng/mg; *p* = 0.0001]; FGF‐23 [1.07 (0.4–32.6) vs. 44.1 (6–162) ng/mg; *p* = 0.01]; and osteoprotegerin [0.16 (0.08–2.4) vs. 6.5 (1.1–23.7) ng/mg; *p* = 0.001]), upregulation of the p53 gene, and downregulation of Dickkopf‐1 gene expression. Patients with high serum A1c levels presented greater cortical porosity and Mlt and reduced osteoblast surface/bone surface, eroded surface/bone surface, osteoclast surface/bone surface, mineral apposition rate, and adjusted area. Cortical thickness was negatively correlated with serum A1c (R = −0.28; *p* = 0.02) and pentosidine levels (R = −0.27; *p* = 0.02). AGEs accumulation in the bone of CKD patients was related to decreased bone protein expression, gene expression changes, and increased skeletal resistance to PTH; A1c and pentosidine levels were related to decreased cortical thickness; and A1c levels were related to increased cortical porosity and Mlt. © 2023 The Authors. *JBMR Plus* published by Wiley Periodicals LLC on behalf of American Society for Bone and Mineral Research.

## Introduction

Mineral and bone disorder (MBD) is a major complication of chronic kidney disease (CKD) and causes systemic effects, resulting in cardiovascular disease, bone fractures, and increased mortality.^(^ ^1^, ^2^, ^3^, ^4^, ^5^ ^)^

MBD pathophysiology is related to the accumulation of many uremic toxins, such as phosphate and parathormone.^(^ ^6^, ^7^ ^)^ Advanced glycation end products (AGEs) constitute one group of uremic toxins, whose effect on bone metabolism in CKD patients is poorly understood.^(^ ^8^, ^9^, ^10^ ^)^

AGEs represent a heterogeneous group of molecules, constituted by nonenzymatic glycation reactions reducing sugars, amino acids, lipids, or DNA. These AGEs molecules accumulate with the CKD progression and activate intracellular signals through nonspecific, specific receptors (RAGEs) and non‐receptor‐mediated mechanisms, leading to increased production of reactive oxygen species and inflammatory cytokines.^(^ ^10^, ^11^ ^)^

At the cellular level, AGEs are related to the decreased differentiation and proliferation of osteoblasts, osteoclasts, and mesenchymal stem cell apoptosis. AGEs also affect matrix protein production and lead to collagen cross‐linking activity alterations.^(^ ^12^, ^13^, ^14^ ^)^ Some studies have identified a link between AGEs, osteoporosis, and bone fractures in clinical settings.^(^ ^15^, ^16^, ^17^ ^)^

However, evidence of the effects in CKD is scarce, and the underlying mechanisms are not fully understood. In a rat model of renal osteodystrophy induced by adenine, Aoki et al. observed a greater accumulation of AGEs in peritrabecular osteoblasts and suppressed expression of runt‐related transcription factor 2 (RUNX2), alkaline phosphatase, secreted phosphoprotein‐1, and lysyl oxidase mRNA levels than in normal animals. The authors suggest that these findings represent suppression of osteoblast differentiation and function.^(^ ^9^ ^)^ Chen et al. tested the effects of AGEs lowering drug ALT‐711 in the aorta and bone. They observed bone AGEs content reduction without any improvement in bone mechanics.^(^ ^10^ ^)^ In humans, Mitome et al. observed a significant presence of pentosidine in bone from patients on dialysis. The pentosidine concentration was inversely related to the bone formation rate and volume.^(^ ^18^ ^)^

Together, these studies reveal the need to deepen knowledge of the effects of AGEs on bone metabolism to generate new hypotheses to better understand their contribution to the pathophysiology of CKD‐MBD. For this reason, we performed an extensive examination of the impact of AGEs in bone from patients with CKD at different stages and treatments. Our working hypothesis is that CKD is associated with the accumulation of AGEs, which in turn results in the dysfunction of bone metabolism, expressed by bone morphological alterations, reduced protein synthesis, and changes in gene expression. Our primary aim was to identify and quantify the accumulation of AGEs and RAGEs in bone and AGEs in the blood (serum pentosidine, carboxymethyl lysine, and glycated hemoglobin levels) and skin and then study the relations between AGEs accumulation, bone histology, protein and gene expression, and serum markers of bone metabolism.

## Material and Methods

### Study design and patient selection

Eighty‐six patients at different CKD stages were enrolled in this observational and double‐center study from February 2016 to November 2017. Patients were recruited from the Nephrology Department's outpatient clinics at the Hospital de Clínicas of the State University of Campinas (UNICAMP) and São Paulo State University (UNESP) and divided into the following CKD subgroups: CKD stages 3–5 nondialysis on conservative management but still without a clinical indication for dialysis (*n* = 26), hemodialysis (HD, *n* = 32), and peritoneal dialysis (PD, *n* = 28). Patients were selected for convenience, sequentially, and according to the established inclusion and exclusion criteria. They were not part of another study, and all tests performed were part of the research protocol provided for this study.

The inclusion criteria were age over 18 years, in CKD stages 3 to 5D according to *Kidney Disease Outcomes Quality (KDIGO)*,^(^ ^19^ ^)^ and, specifically for patients under HD/PD, to be under these treatments for at least 3 months. The CKD‐EPI equation estimated the glomerular filtration rate.^(^ ^20^ ^)^ Patients in the HD subgroup were on chronic HD treatment three times weekly, 4 hours/session, using high‐flux and high‐efficiency polysulfone dialyzers. Patients in the PD subgroup were on automated PD (*n* = 18) or continuous ambulatory PD (*n* = 10).

Exclusion criteria were the presence of chronic inflammatory disease, primary hyperparathyroidism, kidney transplantation, acute cardiovascular event in the 3 months before screening for inclusion, cognitive impairment, cancer, HIV, and clinical instability; 12 (46%) patients in the CKD 3–5 nondialysis subgroup were classified as having CKD stage 3, 11 (42%) stage 4, and three (12%) stage 5 nondialysis. Written informed consent was obtained from all patients; the local ethics committee approved the study protocol under numbers CAAE 38108314.6.0000.5404‐45943115.9.0000.5404‐45777015.5.0000.5404, and the clinical and research activities being reported are consistent with the Declaration of Helsinki.

### Measurement of AGEs levels by skin autofluorescence (AGE‐sAF)

AGE skin deposition was evaluated by SAF using the AGE‐Reader™ (DiagOptics BV, Groningen, the Netherlands). This device measures fluorescence emitted from the skin influenced by the deposition of AGEs, and it calculates the ratio between emitted and reflected excitation light. The measurements were in triplicate on the ventral side of the forearm. Areas with arterial–venous fistulas, scars, and tattoos were avoided. The mean values were used for all statistical analyses and AGEs levels in skin expressed as arbitrary units (AU).

### Measurement of serum AGE levels

In accordance with the manufacturer's instructions, serum pentosidine and N‐carboxymethyl lysine levels were determined by enzyme‐linked immunosorbent assay (ELISA) (pentosidine, kit provided by Cusabio Biotech Co. Ltd.; N‐carboxymethyl lysine, kit supplied by Blue Gene Biotech Co.). The detection ranges of the pentosidine and N‐carboxymethyl lysine kits were 25–2000 pmol/mL and 5–100 ng/mL, respectively.

### Biochemical analysis

Serum intact parathyroid hormone (PTH) levels (reference range: 15–65 pg/mL) were measured using a chemiluminescence assay (Liaison N‐tact PTH CLIAR, Diasorin, Stillwater, USA). Serum 25‐hydroxyvitamin D levels (reference range: 30–100 ng/dL) were measured using a chemiluminescence method. Alkaline phosphatase (reference range: 30–120 IU/L) was measured using a kinetic colorimetric test (Beckman Coulter OSR6104, California, USA). Serum calcium and phosphate levels and a general serum biochemistry profile were assayed by standard autoanalyzer techniques in an on‐site biochemistry laboratory (Modular IIPR system, Roche Diagnostics, Basel, Switzerland). Serum tartrate‐resistant acid phosphatase 5b (TRACP‐5b) levels were determined using an ELISA kit (MicroVue, Quidel, Santa Clara, CA, USA), normal range: 1.2–6.7 U/L; sclerostin was assessed by Teco Sclerostin EIA Kit (enzyme‐linked immunosorbent assay; Teco Medical Group, Sissach, Switzerland, reference range: 0.2–0.6 ng/mL). Intact fibroblast growth factor 23 (FGF‐23), Dickkopf‐related protein 1 (DKK1), and receptor activator of nuclear factor kappa‐B ligand (RANKL) were measured using a multiplex assay kit (Merck Millipore, Darmstadt, Germany). Plasma specimens were prepared for analysis utilizing a multiplex assay kit (Milliplex Human Bone Magnetic Bead Panel, EMD Millipore Corp., Massachusetts, USA) according to specific protocols provided by the company. Blood samples were collected on a previously scheduled date for patients in the CKD 3–5 nondialysis and PD groups. Blood samples of hemodialysis patients were collected immediately before the week's second session.

### Bone biopsy

Bone biopsy samples were obtained from the right or left iliac crests through a trephine for bone biopsy (diameter = 7 mm), adapted to an electrical drill (dewalt™ and Rochester bone trephine™, USA). A double labeling tetracycline course for 3 days (20 mg/kg/d), with a 10‐day interval, was used. The biopsy was performed 3–5 days after the last tetracycline dose. A bone fragment was divided into three parts for histomorphometry, immunohistochemistry, and molecular biology studies. The undecalcified bone fragments were submitted to histological processing and analysis. Bone histomorphometry was performed through a semiautomatic method (Osteomeasure™, Osteometrics, Atlanta, GA, USA). The static, dynamic, and structural histomorphometric indices were reported using international nomenclature.^(^ ^21^ ^)^

### Immunohistochemistry and quantification of accumulation of AGEs and RAGEs expression

Immunohistochemical quantification of the accumulation of AGEs and RAGEs expression in bone was performed by adapting a method previously reported by Gomes.^(^ ^22^ ^)^ In brief, two adjacent 5‐μm sections of bone tissue were placed side by side on each slide. Bone sections were deacrylated in a 1:1 mixture of xylene and chloroform for 30 minutes, rehydrated in graded alcohol solutions, submitted to a quick semidecalcification with 1% acetic acid for 10 minutes, and rinsed twice with distilled water. Endogenous peroxidase activity was inhibited by a mixture of 3% hydrogen peroxide in methanol for 30 minutes, followed by two water washes. The samples were incubated with protein block (DakoCytomation, California, USA) to block nonspecific binding. Sections were incubated overnight at 4°C in a humidified chamber using the primary rabbit polyclonal antibodies anti‐AGE (ab23722, Abcam, Cambridge, UK) (dilution 1:5000) and anti‐RAGE (16346‐1‐AP, Proteintech Group, Manchester, UK) (dilution 1:200). After incubation with the secondary antibody, the slides were incubated with the avidin/biotin HRP complex. The revelation was developed with a Vector kit (DAB Substrate Kit, Vector Laboratories, Burlingame, USA) (1 drop DAB +1 mL of the substrate). The sections were rinsed in distilled water and counterstained with Mayer's hemalum solution (Merck KGaA, Darmstadt, Germany). Negative controls were performed by omitting the primary antibody. For analysis, the images were captured using an Olympus BX53 photomicroscope (Olympus Corp., Tokyo, Japan) integrated into a computer and analyzed using Image‐Pro Premier® software (Media Cybernetics, Rockville, USA). The entire extent of the trabecular and cortical bone tissue regions were photographed with final magnifications of ×100 and ×400, and through the software, the areas of interest imunostained for anti‐AGEs and anti‐RAGEs were automatically quantified through the creation of a macro capable of identifying in each pixel of the image the shade of the brown color defined as being representative of positive immunostaining, in relation to the negative control. The immunopositivity was expressed as a percentage of the total software‐classified areas.

### Quantification of bone protein by multiplex

Protein lysates were extracted from bone samples. The bone contents of sclerostin, osteocalcin, DKK1, and FGF‐23 were measured with a multiplex assay kit (Human Bone Magnetic Bead Panel, Milliplex, EMD Millipore Corp., Darmstadt, Germany) based on Luminex™ xMAP technology according to the manufacturer's instructions.

### Gene expression

After total RNA extraction from bone sample using trizol, a NanoDrop 1000 spectrophotometer (Thermo Scientific) was used to determine the total RNA amount. cDNA was synthesized from total RNA by reverse transcriptase (Improm‐II Reverse Transcriptase, Promega Corp., Madison, WI, USA) using a thermocycler (DNA Engine, MJ Research, Massachusetts, USA).

Gene expression was determined from the cDNA through quantitative PCR using SYBR Green (Rotor‐Gene SYBR Green PCR kit, Qiagen, Hilden, Germany) and a Rotor‐Gene Q thermocycler (Qiagen, Hilden, Germany). The genes analyzed were SOST (AF_331844.1), RANKL (NM_003701.3), OPG (U94332), β‐catenin (X_87838.1), FGF‐23 (NM_020638.2), p53 (NM_001276760), DKK (NM_012242.4), Osterix (AF477981), ALP‐1 (J04948.1), collagen 1 (D21337.1), BGLAP (NM_199173), and the reference gene GAPDH (glyceraldehyde 3‐phosphate dehydrogenase‐NM_002046.4) with their respective primers designed from IDT (Integrated DNA Technologies, Coralville, USA). Gene expression was calculated by the ΔΔCt method of relative quantification. Values are expressed as a multiple (fold) of the expression compared to the value of the calibrator. The statistical analysis between groups was performed using REST™ software (Qiagen, Hilgen, Germany).

### Statistical analysis

The continuous variables are reported as the mean ± SD or medians and interquartile intervals. Categorical data are reported as frequencies and percentages. Comparisons between the continuous variables, skewed data, and categorical variables were performed using the Student's *t* test, the Mann–Whitney test, and the chi‐square test. To detect associations between AGEs accumulation and changes in bone metabolism, the median value of AGEs accumulation parameters (glycated hemoglobin, N‐carboxymethyl lysine, pentosidine, SAF, and AGEs/RAGEs accumulation/expression in bone) was used for the purpose of comparison. Spearman's coefficient test provided the significance of the correlation between noncontinuous variables. Statistical analyses were performed using SPSS 22.0 (SPSS, Chicago, IL, USA). A two‐sided *p*‐value <0.05 was considered statistically significant.

## Results

### General findings and accumulation of AGEs in serum, skin, and bone

We considered data from 86 individuals for analysis; this was a population with a mean age of 51 ± 13 years; 48 (56%) were male, 41 (48%) were Caucasian, and 16 (19%) had type 2 diabetes. All participants had CKD; 32 (37%) were on HD, 28 (33%) were on PD, and 26 (30%) were on conservative management, displaying an estimated glomerular filtration rate of 26.9 (17.2–34.5) mL/min/1.73 m^2^. The baseline characteristics of the study population are summarized in Table 1. Clinical, demographic, and biochemistry findings of all CKD populations and subgroups are summarized in supplementary data (Table S1).

**Table 1:** General Clinical and Biochemical Data

*N* = 86	
Age (years)	51 ± 13
Male (*N*, %)	48 (56)
Caucasian (*N*, %)	41 (48)
Etiology of chronic kidney disease (*N*, %)	
Hypertension	23 (27)
Chronic glomerulonephritis	16 (19)
Diabetes mellitus	9 (10)
Dialysis vintage (months)	21 (10–44)
Body mass index (kg/m^2^)	26 ± 4.8
Hemoglobin (g/dL)	12.1 (11–13.6)
Albumin (g/dL)	3.7 (3.3–4.0)
Total calcium (mg/dL)	8.9 ± 0.8
Phosphate (mg/dL)	5 ± 1.6
25‐vitamin D (ng/mL)	28.1 (20.8–34.5)
FGF‐23 (ng/mL)	1570 (273–6499)
Sclerostin (ng/mL)	1.46 (0.94–2.19)
Alkaline phosphatase (IU/mL)	90 (71–112)
Parathormone (pg/mL)	228 (117–439)
RANKL (pg/mL)	0.19 (0.01–0.75)
TRACP‐5b (U/L)	5.1 (3.3–7.7)

Abbreviations: FGF‐23, fibroblast growth factor‐23; RANKL, receptor activator of nuclear factor kappa‐Β ligand; TRACP‐5b, tartrate‐resistant acid phosphatase 5b.

AGEs were detected in blood, skin, and bone in all patients. No correlation was found between measurements of AGEs in blood, skin, and bone.

In serum, the median levels of pentosidine, N‐carboxymethyl lysine, and glycated hemoglobin were 71.6 (44.2–121.2) pmol/mL, 15.2 (9.7–32.4) ng/mL, and 5.4% (5–6.1%), respectively. In skin, the median value of SAF was 3.05 (2.5–3.4) AU and was positively correlated with age (R = 0.55; *p* = 0.0001) and dialysis vintage (R = 0.30; *p* = 0.04).

**Fig. 1:**
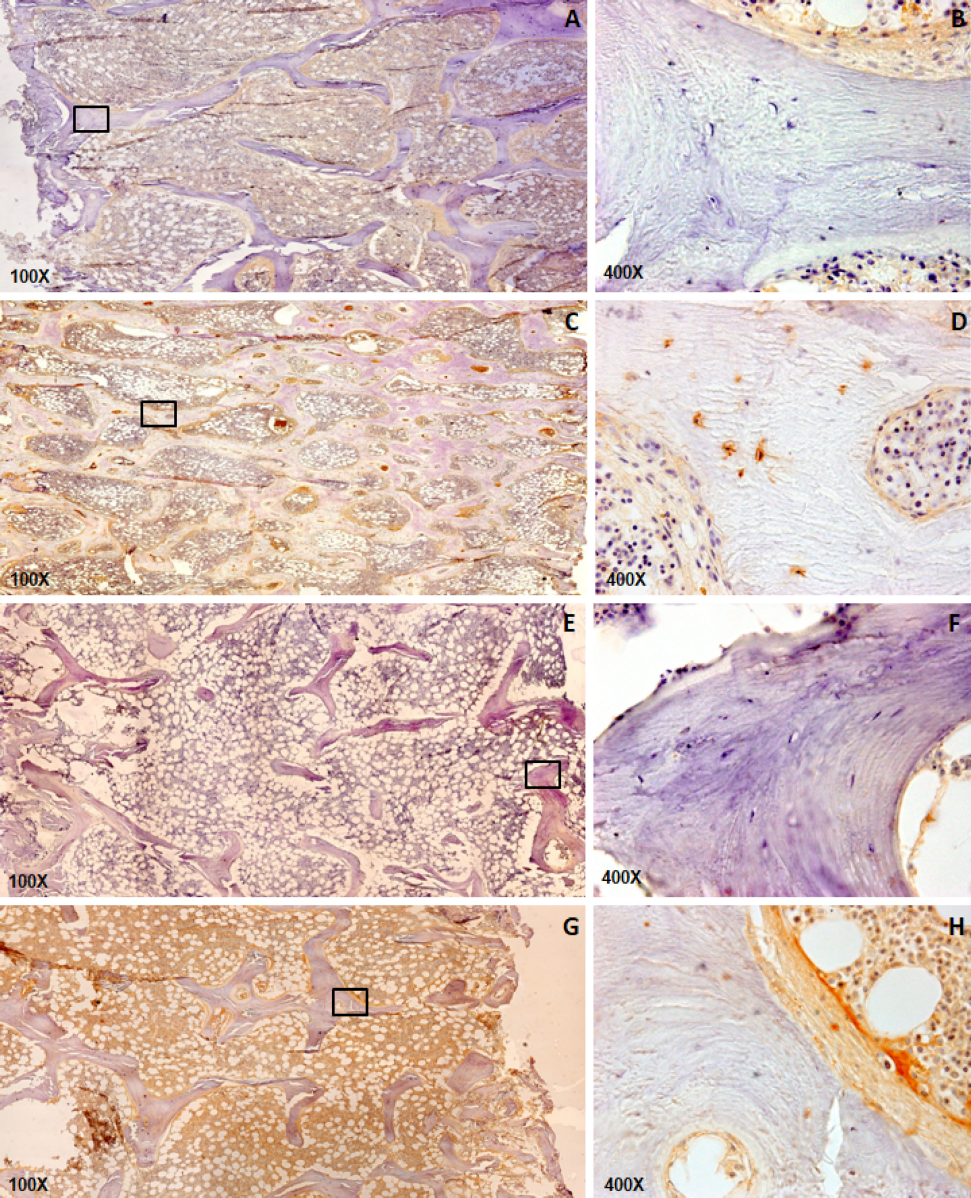
Trabecular and cortical bone AGEs accumulation and RAGEs expression in patients with CKD (AGEs accumulation (*A–D*); RAGEs expression (*E–H*).

In bone, accumulation of AGEs and expression of RAGEs were detected in both trabecular and cortical surfaces in all patients. AGEs in trabecular and cortical bone covered 3.92% (1.6–15.3%) and 5.42% (3–12.1%) of its surface, respectively. RAGE expression in trabecular and cortical bone covered 0.7% (0.13%–2.88%) and 0.83% (0.2–2.3%) of its surface, respectively (Fig. 1*A–H*). Of note, AGEs accumulation seems to demonstrate affinity with osteocytes (Fig. 1, detail *D*).

AGEs in trabecular bone were positively correlated with AGEs in cortical bone (R = 0.77; *p* = 0.0001) and dialysis vintage (R = 0.31; *p* = 0.03) and negatively correlated with the serum RANKL/PTH ratio (R = −0.25; *p* = 0.03).

RAGEs expression in trabecular bone was positively correlated with RAGEs expression in cortical bone (R = 0.76; *p* = 0.0001), dialysis vintage (R = 0.49; *p* = 0.03), phosphate (R = 0.26; *p* = 0.03), and parathormone (R = 0.40; *p* = 0.001) and negatively correlated with serum glycated hemoglobin levels (R = −0.26; *p* = 0.03) and the TRACP‐5b/PTH ratio (R = −0.31; *p* = 0.01).

### Serum and skin AGEs levels: associations with bone morphology and metabolism

Patients presenting high serum glycated hemoglobin levels displayed greater cortical porosity [1.9 (1.2–3.1) vs. 1.18 (0.47–2.22); *p* = 0.02], mineralization lag time [24.8 (18–54.2) vs. 19.1 (9.8–34.7); *p* = 0.03], reduced osteoblast surface/bone surface [1.7 (1–3.6) vs. 3.6 (1.4–6.7); *p* = 0.04], eroded surface/bone surface [2.4 (1.5–3.9) vs. 4.9 (3.2–7.9); *p* = 0.0001], osteoclast surface/bone surface [0.1 (0.04–0.18) vs. 0.29 (0.11–0.52); *p* = 0.0001], mineral apposition rate [0.59 (0.47–0.71) vs. 0.78 (0.48–0.98); *p* = 0.02] and adjusted apposition rate/bone area [0.26 (0.12–0.42) vs. 0.38 (0.25–0.68); *p* = 0.009]. Cortical thickness was negatively correlated with serum glycated hemoglobin (R = −0.28; *p* = 0.02) and with pentosidine levels (R = −0.27; *p* = 0.02). No differences were observed in bone parameters according to median levels of N‐carboxymethyl lysine.

No differences in bone protein expression were observed according to serum AGEs levels. Bone histomorphometric parameters, bone protein, and gene expression were similar among groups defined by the median skin AGEs accumulation.

### Bone AGEs accumulation and RAGEs expression: associations with bone morphology and metabolism

Bone histology was similar among groups defined by the median AGEs accumulation in trabecular bone (Table 2). Patients with high levels of AGEs in trabecular bone had decreased bone levels of sclerostin [1.96 (0.11–40.3) vs. 89.3 (2.88–401) ng/mg; *p* = 0.004], DKK1 [0.064 (0.03–0.46) vs. 1.36 (0.39–5.87) ng/mg; *p* = 0.0001], FGF‐23 [1.07 (0.4–32.6) vs. 44.1 (6–162) ng/mg; *p* = 0.01] and osteoprotegerin [0.16 (0.08–2.4) vs. 6.5 (1.1–23.7) ng/mg; *p* = 0.001] compared with patients presenting low trabecular bone AGEs levels (Table 3). Above‐median trabecular bone AGEs accumulation upregulated the p53 gene and downregulated DKK1 gene expression. Comparisons of bone gene expression according to median of accumulation of AGEs in skin, trabecular bone and expression of RAGEs in trabecular bone are summarized in supplementary data (Table S2).

**Table 2:** Histomorphometric Bone Parameters According to Median AGEs Accumulation and RAGEs Expression in Trabecular Bone

	Accumulation of AGEs in trabecular bone (%)			RAGEs expression in trabecular bone (%)		
	<3.92	≥3.92	*p*	<0.707	≥0.707	*p*
BV/TV (%)	18.8 (16–28)	23.9 (19.3–29)	0.09	18.9 (15.4–24.4)	26.2 (19.2–30.7)	**0.002**
Tb.Th (μm)	123 (104–145)	132 (118–147)	0.2	118 (107–136)	135 (125–154)	**0.003**
Tb.Sp (μm)	478 (383–591)	430 (363–568)	0.22	531 (410–595)	398 (316–531)	**0.016**
Tb.N (mm/mm)	1.6 (1.4–1.9)	1.8 (1.38–2.1)	0.21	1.5 (1.4–1.9)	1.8 (1.5–2.1)	**0.04**
OV/BV (%)	1.75 (0.6–3.2)	1.15 (0.6–2.1)	0.2	1.19 (0.6–2.2)	1.42 (0.6–2.7)	0.54
O.Th (μm)	7.2 (5.5–8.3)	6.3 (5.2–7.5)	0.16	6 (5.2–7.7)	6.9 (5.9–8.5)	0.12
OS/BS (%)	15.1 (6.7–23.6)	10.8 (7–19.7)	0.32	10.9 (6.6–20.8)	12.7 (7.0–23.1)	0.70
Ob.S/BS (%)	1.51 (1.05–4.38)	2.23 (1.36–6.5)	0.13	1.49 (0.97–3.31)	2.38 (1.38–6.31)	**0.05**
ES/BS (%)	3.06 (2.08–4.39)	4.03 (2.14–6.37)	0.23	2.83 (1.99–4.98)	3.73 (2.21–6.58)	0.22
Oc.S/BS (%)	0.16 (0.09–0.38)	0.10 (0–0.29)	0.053	0.1 (0.04–0.20)	0.21 (0.05–0.47)	0.06
MS/BS (%)	5.49 (3.81–10.56)	5.41 (2.97–10.09)	0.78	4.48 (2.00–9.06)	5.81 (3.96–11.8)	0.21
MAR (μm/dia)	0.64 (0.48–0.92)	0.63 (0.47–0.74)	0.53	0.62 (0.47–0.72)	0.64 (0.48–0.94)	0.32
BFR/BS (μm^3^/μ^2^/d)	0.041 (0.019–0.092)	0.035 (0.225–0.558)	0.61	0.03 (0.015–0.054)	0.0492 (0.027–0.098)	0.08
Aj.Ar (μm/d)	0.29 (0.13–0.63)	0.3 (0.22–0.55)	0.67	0.27 (0.14–0.52)	0.36 (0.2–0.57)	0.28
Mlt (d)	23.7 (11.5–54.3)	19.8 (12.1–35.2)	0.4	22.1 (13.2–45.3)	19.8 (10.8–47.6)	0.58
Fb.V/TV (%)	0.03 (0.01–0.14)	0.03 (0.006–0.13)	0.89	0.02 (0.003–0.05)	0.04 (0.11–0.29)	**0.04**
Ct.V/BV (μm^3^)	22.5 (13.1–28)	21.3 (17–26)	0.95	21.7 (12.2–28)	21.4 (16.1–25.8)	0.80
Ct.Th (μm)	626 (423–782)	627 (499–729)	0.78	606 (416–723)	630 (514–779)	0.33
Ct. Po (%)	1.89 (1.1–2.8)	1.4 (0.7–2.7)	0.44	1.71 (1.03–2.83)	1.27 (0.77–2.55)	0.44

Abbreviations: Aj.Ar, adjusted area; BFR/BS, bone formation rate/bone surface; BV/TV, bone volume/tissue volume; Ct.Po, cortical porosity; Ct.Th, cortical thickness; Ct.V/BV, cortical volume/bone volume; ES/BS, eroded surface/bone surface; Fb.V/TV, fibrose volume/tissue volume; MAR, mineral apposition rate; Mlt, mineralization lag time; MS/BS, mineralized surface/bone surface; O.Th, osteoid thickness; Ob.S/BS, osteoblast surface/bone surface; Oc.S/BS, osteoclast surface/bone surface; OS/BS, osteoid surface/bone surface; OV/BV, osteoid volume/bone volume; Tb.Sp, trabecular separation; trabecular number; Tb.Th, trabecular thickness.

**Table 3:** Comparisons of Bone Protein Expression Based on Median of Trabecular Bone AGEs Accumulation or RAGEs Expression

	AGEs accumulation in trabecular bone (%)			RAGEs expression in trabecular bone (%)		
	<3.92	≥3.92	*p*	<0.707	≥0.707	*p*
Sclerostin (ng/mg)	89.3 (2.88–401.2)	1.96 (0.11–40.3)	**0.004**	38.5 (0.37–344.7)	19.9 (0.53–196)	0.944
DKK1 (ng/mg)	1.36 (0.39–5.87)	0.064 (0.03–0.46)	**0.0001**	0.51 (0.06–4.46)	0.77 (0.06–3.11)	0.907
FGF‐23 (ng/mg)	44.1 (6–161.9)	1.07 (0.4–32.6)	**0.012**	13.0 (0.7–151)	27.4 (0.8–106)	0.851
Osteoprotegerin (ng/mg)	6.49 (1.13–23.7)	0.16 (0.08–2.4)	**0.001**	2.4 (0.14–22.2)	2.45 (0.18–14.7)	0.963
Osteocalcin (μg/mg)	172.4 (35–439.7)	225.9 (74.6–497.3)	0.593	177 (82–420)	147 (42.9–733)	0.814

Abbreviations: FGF‐23, fibroblast growth factor‐23.

Patients above the median of trabecular RAGEs expression had increased bone volume/tissue volume [26.2% (19.2–30.7%) vs. 18.9% (15.4–24.4%); *p* = 0.002], trabecular thickness [135 (125–154) vs. 118 (107–136) μm; *p* = 0.003], trabecular number [1.83 (1.5–2.1) vs. 1.5 (1.4–1.9) mm/mm; *p* = 0.04], and fibrosis volume/bone volume [0.04% (0.11–0.29%) vs. 0.02% (0.003–0.05%); *p* = 0.04], and decreased trabecular separation [398 (316–531) vs. 531 (410–595) μm; *p* = 0.02] (Table 2).

Bone proteins and gene expression did not reveal differences according to the median trabecular bone RAGEs expression (Table 3). Patients above the median of trabecular bone RAGEs expression had increased serum levels of parathormone (277 [152–416] vs. 206 [71–456] pg/mL; *p* = 0.01) and FGF‐23 (1431 [400–7319] vs. 1120 [123–9985] ng/mL; *p* = 0.003).

### Treatment regimen

To evaluate differences across treatment regimens regarding AGEs levels, we compared individuals on HD, PD, and conservative treatment. Only a few differences were noted between the treatment regimens.

N‐Carboxymethyl lysine levels were higher in PD patients than HD and conservative patients (25 [12–52] vs. 13 [9–22] and 10 [9–25] ng/mL, respectively; *p* = 0.009). Serum glycated hemoglobin levels were higher in HD than in PD and conservative treatment (5.9% [5.5–6.7%] vs. 5.7% [5.1–6.2%] vs 5.2% [5–5.4%], respectively; *p* = 0.0001). RAGEs expression in trabecular bone was higher in HD than in conservative treatment (1.21 [0.23–4.67] vs. 0.18 [0.06–1.44]; *p* = 0.030), whereas no differences across groups were reported for cortical bone. No difference was found among groups regarding serum pentosidine levels, AGEs in skin, AGEs in bone surface, and RAGEs expression in cortical bone.

## Discussion

Our study confirmed that bone AGEs accumulation occurred in patients with CKD and might be an early event in the CKD course since no significant differences in their levels were noted across distinct CKD stages or treatments. The AGEs accumulation in bone was related to decreased bone protein expression and changes in gene expression. The relationship observed between bone AGEs accumulation and a decreased serum RANKL/PTH ratio suggests that AGEs contribute to skeletal resistance to the actions of PTH. Although bone and skin AGEs accumulation were not related to significant histological changes, serum pentosidine and glycated hemoglobin levels were related to decreased cortical thickness; glycated hemoglobin levels were related to increased cortical porosity and mineralization lag time. Trabecular bone RAGEs expression was related to better structural bone parameters.

As far as we know, this is the first study to reveal bone AGEs accumulation along CKD stages and treatments and its impact on histology, bone protein, and gene expression. In animals with CKD, Aoki et al. detected AGEs in peritrabecular osteoblasts by immunohistochemistry and western blot techniques.^(^ ^9^ ^)^ In humans with CKD, pentosidine‐induced cross‐links by HPLC were detected in the bones of 21 patients under HD and one in PD. The authors observed a negative correlation between pentosidine in bone and bone‐formation rate (BFR)/bone volume (BV) and MAR, but the number of subjects in this analysis was limited to 10.^(^ ^18^ ^)^

Our findings reveal a relation between bone AGEs accumulation and a marked reduction in key bone protein expression, namely, osteoprotegerin, FGF‐23, sclerostin, and DKK1. Osteoprotegerin, also known as osteoclastogenesis inhibitory factor, is expressed by osteoblasts and plays a central role in regulating bone mass. Osteocytes and osteoblasts mainly secrete FGF‐23 in bone, and studies have shown that FGF‐23 overexpression or suppression is associated with defects in skeletal mineralization.^(^ ^23^, ^24^, ^25^ ^)^ Sclerostin and DKK1 are Wnt/β‐catenin pathway inhibitors and are considered negative regulators of bone mass. Sclerostin is produced mainly by osteocytes, while DKK1 is mainly produced by osteoblasts.^(^ ^26^, ^27^ ^)^ It is worth highlighting that bone AGEs accumulation was detected mainly around osteocytes and osteoblasts (Fig. 1*D–H*). The reduction in proteins synthesized by osteocytes and osteoblasts maybe reflects the important dysfunction of these cells due, at least partially, to AGEs accumulation.^(^ ^12^, ^13^ ^)^ The reduction in bone protein expression, such as FGF‐23 and sclerostin, was observed based on intragroup comparison according to the median AGEs accumulation in trabecular bone. However, we do not know whether there is a certain cut‐off level related to the reduction expression of these proteins after which the circulating levels of these molecules would be affected. As expected in CKD patients, in our study we observed elevated serum levels of FGF‐23 and sclerostin, regardless of the level of AGEs accumulation in trabecular bone or protein expression.

Bone gene expression and regulation are complex processes, and scientific reports about AGEs and bone genes in patients with CKD is scarce.^(^ ^28^, ^29^, ^30^, ^31^, ^32^ ^)^ In our study, we observed that AGEs may upregulate p53 and downregulate DKK1 gene expression in patients who presented above‐median trabecular bone AGEs accumulation.

p53 is a well‐known tumor suppressor that promotes cell cycle arrest, programmed cell death, and cell senescence and acts as a transcriptional repressor.^(^ ^29^ ^)^ Verma et al. observed that AGEs impair the autophagy process in p53‐negative cells and then promote apoptosis via regulation of NF‐κB. The authors claim that p53 acts antagonistically to prevent this impairment.^(^ ^30^ ^)^ It is plausible to think that this mechanism may explain our cohort's observed upregulation of the p53 gene.

In contrast to our findings about AGEs‐mediated downregulation of DKK1 gene expression, Li et al. observed an AGEs‐induced inhibition of the Wnt/β‐catenin pathway in vitro.^(^ ^31^ ^)^ Notsu et al. demonstrated that incubating osteocyte‐like cells with AGEs increased the sclerostin‐producing gene Sost transcription in a dose‐dependent manner.^(^ ^32^ ^)^ Both studies were performed under controlled conditions, while our data were from patients. This contrasting finding probably occurs because regulation of bone metabolism depends on several factors in complex systems. Other factors that alter sclerostin or DKK1, such as parathormone, FGF‐23, or even AGEs, may play a role.^(^ ^33^, ^34^ ^)^

Tominaga et al. examined factors related to bone responsiveness to PTH in patients undergoing chronic hemodialysis. They proposed the TRACP‐5b/intact PTH (iPTH) ratio as an index that reflects bone responsiveness to PTH.^(^ ^35^ ^)^ In our cohort, we observed that patients with an AGEs accumulation and RAGEs expression in trabecular bone above the median presented decreased RANKL/PTH and TRACP‐5b/PTH ratios, respectively. These findings suggest that AGEs could be another factor for skeletal resistance to PTH in patients with CKD.

Our study found no correlation between serum, skin, and bone levels of AGEs. This finding agrees with previous observations; in human body tissues, organs and structures seem to present different affinities for AGEs accumulation, either by constitution itself as due to metabolism particularities. For example, the proteins in the human eye are highly susceptible to the formation of AGEs, which accumulate at a higher rate in diseases such as cataracts. As bone turnover is lower than other tissues, some authors hypothesized that bone was potentially more susceptible to AGEs accumulation and effects. AGEs measurement in serum samples remains a challenge because of the lack of standardized methods and because circulating AGEs may not accurately reflect their accumulation in body tissues, which results from long‐term exposure. Skin measurement using SAF attenuates this effect, since AGEs accumulation in the skin may be more closely related to AGEs deposition in the bone, yet the differences affect the correlation between these events in the intracellular synthesis of AGEs that vary across tissues.^(^ ^36^, ^37^, ^38^, ^39^ ^)^

Our results showed that serum pentosidine and glycated hemoglobin levels were related to decreased cortical thickness, increased cortical porosity, and mineralization lag time. We observed that glycated hemoglobin affected cortical bone differently than pentosidine. Previous studies in animals and humans showed direct relationships between glycated hemoglobin and cortical microarchitecture alteration.^(^ ^40^, ^41^ ^)^ However, Sroga et al. observed differences in the progression of bone pathologies related to protein glycation by different sugars: in vitro glycation of bone using glucose leads to the formation of lower levels of AGEs, whereas ribosylation appears to support a pathway toward pentosidine formation.^(^ ^42^ ^)^ This observation suggests differential actions of different AGEs in cortical bone.

This study had limitations. It was an observational and, essentially, descriptive study able to generate new hypotheses. The conclusions suggest relationships between bone AGEs accumulation and a decrease in essential bone proteins, changes in bone gene expression, potential increased skeletal resistance to PTH, and negative effects of serum AGEs on cortical bone. To overcome the lack of a control group, we attempted to perform statistical analyses through intragroup comparisons of AGEs accumulation parameters according to their respective medians; since there is no standardization of serum pentosidine and CML levels measurements at CKD setting, as well comparisons with more accurate methods such as HPLC, the results on these parameters must be interpreted with caution; we cannot exclude the possibility that the lack of correlation between AGEs accumulation in trabecular bone and BFR/bone surface (BS) was modified by serum PTH levels due to potential sample selection bias. Further interventional studies are required to confirm the mechanisms involved in these associations.

Our study also had some strengths. Most importantly, (i) it demonstrated, using bone biopsy, that AGEs and RAGEs accumulate in the bone of CKD subjects; (ii) a myriad of morphofunctional and genetic parameters was analyzed, providing insightful data on the mechanisms involved in renal osteodystrophy; and (iii) skin, serum, and bone AGEs levels were evaluated, and their associations with bone metabolism impairment were explored.

## Conclusions

We demonstrated that bone accumulation of AGEs occurs in patients with CKD and might affect the metabolism of this tissue. A possible mechanism would be a reduction in the synthesis of essential bone proteins and changes in gene expression. Cortical bone seems to be affected by different serum AGEs. The mechanisms behind these interactions, including differential effects according to AGEs types, should be explored in specific studies. Reducing AGEs accumulation may be a therapeutic target that would modify the progression of skeletal disorders in patients with CKD.^(^ ^10^ ^)^ Pharmacological studies or dietary approaches should be proposed to test the effects of lowering AGEs on outcomes involving skeletal disease caused by CKD.

## Author Contributions

**Kélcia R. S. Quadros:** Conceptualization; data curation; formal analysis; investigation; writing – original draft; writing – review and editing. **Noemí A. V. Roza:** Data curation; formal analysis; methodology; writing – review and editing. **Renata A. França:** Investigation; writing – review and editing. **André B. A. Esteves:** Investigation; writing – review and editing. **Joaquim Barreto:** Formal analysis; methodology; writing – original draft; writing – review and editing. **Wagner V. Domingues:** Formal analysis; methodology; writing – review and editing. **Luzia N. S. Furukawa:** Formal analysis; methodology; writing – review and editing. **Jacqueline Teixeira Caramori:** Investigation; writing – review and editing. **Andrei C. Sposito:** Conceptualization; writing – review and editing. **Rodrigo Bueno de Oliveira:** Conceptualization; data curation; formal analysis; funding acquisition; project administration; resources; supervision; writing – original draft; writing – review and editing.

## Conflict of Interest

The authors declare there are no competing financial interests.

### Peer Review

The peer review history for this article is available at https://publons.com/publon/10.1002/jbm4.10727.

## Supporting information


**Table S1.** Clinical, demographic, and biochemistry findings of all CKD populations and subgroups.
**Table S2.** Comparisons of bone gene expression according to median of accumulation of AGEs in skin, trabecular bone, and expression of RAGEs in trabecular bone.

## Data Availability

The data that support the findings of this study are available from the corresponding author upon request.
