# Supplementary material for: Advanced Glycation End Products and Bone Metabolism in Patients with Chronic Kidney Disease
Source: JBMR Plus. 2023 Feb 16;7(3):e10727. doi: 10.1002/jbm4.10727 (PMC10020922; doi:10.1002/jbm4.10727)
Supplement: Supplementary file 1 — Table S1. Clinical, demographic, and biochemistry findings of all CKD populations and subgroups. Table S2. Comparisons of bone gene expression according to median of accumulation of AGEs in skin, trabecular bone, and expression of RAGEs in trabecular bone. [file JBM4-7-e10727-s001.docx]

**SUPPLEMENTARY DATA**

Table S1. Clinical, demographical and biochemistry findings of all CKD populations and subgroups.

All CKD (N=86) CKD 3-5 (N=26) HD (N=32) PD (N=28) P

Age (years) 51 ± 13 54 ± 12 48 ± 13 51 ± 13 0.28

Male (N, %) 48 (56) 17 (65) 20 (62) 11 (39) 0.09

Caucasian (N, %) 41 (48) 14 (54) 15 (47) 12 (43) 0.2

Etiology of CKD (N, %) **0.005**

Hypertension 23 (27) 9 (35) 11 (34) 3 (11)

Glomerulonephritis 16 (19) 2 (8) 8 (25) 6 (21)

Diabetes *mellitus* 9 (11) 2 (8) 0 (0) 7 (25)

eGFR (mL/min) 6 (5-17) 27 (17-35)^+^ 5 (4-7) 5 (4-7) **0.0001**

Dialysis vintage (months) 21 (10-44) NA 23 (10-68) 17 (10-30) 0.1

Body mass index (kg/m^2^) 26 ± 5 28 ± 5^+^ 25 ± 5 25 ± 4 **0.003**

CML 15.2 (9.7-32) 10.3 (8.7-25.4) 13.2 (9-22) 24.6 (12.1-52.3)* **0.01**

Pentosidine 71.6 (44.2-121) 97.3 (52.5-140) 51.2 (27.8-104) 73.2 (51.4-121) 0.71

Glycated hemoglobin 5.4 (5-6.1) 5.9 (5.5-6.7) 5.2 (5-5.4)** 5.7 (5.1-6.2) **0.001**

Skin AF (AU) 3.1 (2.5-3.4) 3.1 (2.5-3.6) 2.9 (2.4-3.3) 3.1 (2.6-3.5) 0.28

Hemoglobin (g/dL) 12.1 (11-13.6) 14 (12-16)^+^ 11.6 (10.8-12.8) 11.8 (10.4-12.4) **0.0001**

Albumin (g/dL) 3.7 (3.3–4) 3.9 (3.7-4.2) 3.9 (3.6-4.1) 3 (2.6-3.4)^++^ **0.0001**

Total calcium (mg/dL) 8.9 ± 0.8 9.2 ± 0.5 9.1 ± 0.7 8.4 ± 1^++^ **0.0001**

Phosphate (mg/dL) 5.0 ± 1.6 3.6 ± 0.8^+^ 5.9 ± 1.8 5.3 ± 1 **0.0001**

25-vitamin D (ng/dL) 28.1 (21-34) 28.1 (21-34) 31.2 (27.2-36.6) 24.8 (18.9-29.6)^*^ **0.01**

ALP (IU/L) 90 (71-112) 78 (60-101) 95 (72-134) 92 (72-104) 0.1

Parathormone (pg/mL) 228 (118-439) 115 (52-195)^+^ 372 (187-874) 292 (184-536) **0.0001**

Sclerostin 1.46 (0.9-2.2) 0.86 (0.57-1.13)^+^ 1.78 (1.33-2.68) 1.44 (1.12-2.67) **0.001**

FGF-23 (ng/mL) 1570 (273–6499) 51 (18-362) 5689 (1549-88241)**1633 (781-3464) **0.04**

RankL (pg/mL) 0.19 (0.01–0.75) 0.48 (0.01-26.7) 0.19 (0.01-0.48) 0.19 (0-1.17) 0.4

Trap-5b (U/L) 5.01 (3.26-7.68) 4.3 (2.3-5.7) 6.4 (3.9-8.2) 5.1 (3.9-8.5) 0.39

CKD, chronic kidney disease; HD, hemodialysis; PD, peritoneal dialysis; eGFR, estimated glomerular filtration rate; AF, autofluorescence; CML, N-Carboxymethyl lysine; FGF-23, fibroblast growth factor; ALP, alkaline phosphatase; RankL, receptor activator of nuclear factor kappa-Β ligand; TRAP-5b, tartrate-resistant acid phosphatase 5b.

^+^, P < 0.05 for CKD 3-5 *vs*. HD and PD;

*, P < 0.05 for PD *vs*. HD.

**, P < 0.05 for HD vs. CKD 3-4 and PD;

^++^, P < 0.05 for PD *vs*. CKD 3-4 and HD.

Table S2. Comparisons of bone gene expression according to median of accumulation of AGEs in skin, trabecular bone and expression of RAGEs in trabecular bone.

SOST DKK-1 β-Catenin FGF-23 Osterix P53 RANK RANKL OPG γGC Collagen

AGEs in skin ≥ 3.05 AU - - - - - - - - - - -

Trabecular bone

AGEs accumulation ≥ 3.92% **-** ↓ **- - -** ↑ **- - - - -**

RAGEs expression ≥ 0.7 % - - - - - - - - - - -

**↑**, up-regulation; **↓**, down-regulation; AU, arbitrary units; SOST, sclerostin; DKK-1, Dickkopf-1; FGF-23, fibroblast growth factor-23; RANK, receptor activator of nuclear factor kappa-Β; RANKL, receptor activator of nuclear factor kappa-Β ligand; OPG, osteoprotegerin; γGC, gamma-glutamyl carboxylase; Collagen, collagen type-1.
